# VEGFR1‐mediated neuronal cell death following Aß1‐42 oligomers treatment‐ Implications in AD pathogenesis

**DOI:** 10.1002/alz.094971

**Published:** 2025-01-09

**Authors:** Pritam Das

**Affiliations:** ^1^ Mayo Clinic, Jacksonville, FL USA

## Abstract

**Background:**

Recent transcriptome analysis has demonstrated increased expression of Vascular Endothelial Growth Factor receptor‐1 (VEGFR‐1/*FLT1*) and decreased expression of VEGFR‐2/*KDR* in AD brain. Increased expression of VEGFR‐1 and its ligand VEGFB were associated with a more rapid rate of cognitive decline, providing evidence of a potential link between aberrant VEGFR‐1 expression in AD pathogenesis. *In this study, we explored the potential role of aberrant VEGFR‐1 expression in neurons on AD pathology*.

**Method:**

To confirm VEGFR‐1 expression in AD brains, we performed immunostaining in AD brain sections (AD *n* = 5 (Braak stage V‐VI, and normal controls *n* = 5 Braak 0‐II) in AD brains. To determine a potential detrimental role of neuronal VEGFR‐1 expression on AD associated pathologies, we exposed SH‐SY5Y human neuroblastoma cells and mouse primary neurons to either hypoxia conditions (1%O_2_) or 5uM Aß 1‐42 oligomers for 72hrs.

**Result:**

We found increased VEGFR‐1 immunoreactivity in dystrophic neurites in the vicinity of Thio‐S positive “cored” amyloid plaques, whereas VEGFR‐2 antibody staining showed minimal neuronal staining in AD samples. And treatment with hypoxia or Aß oligomers both in SH‐SY5Y cells and mouse primary neurons showed increased VEGFR‐1 expression and cleaved caspase 3 activation**,** leading to neuronal cell death. Significantly, siRNA mediated knockout of VEGFR‐1 in the neurons prevented both hypoxia and Aß oligomer induced cell death. We also show that treatment with either hypoxia or Aß oligomers reduced mRNA expression of cell survival genes including Hippo pathway YAP1 and VEGFR‐2, and siRNA mediated VEGFR‐1 knockdown normalized both YAP1 and VEGFR‐2 levels. Significantly, over‐expression of the VEGFR‐1 by itself reduced mRNA expression of YAP1 and VEGFR‐2 and readily induced neuronal cell death, implicating the VEGFR‐1/VEGFR‐2/YAP1 signaling axis in hypoxia and Aß oligomer induced neuronal cell death, *which is a novel finding*.

**Conclusion:**

These results demonstrate that VEGFR‐1 may play an important transcriptional regulatory role in neuronal cell survival responses and suggest that modulating VEGFR‐1 expression and signaling could have beneficial effects in AD pathogenesis.

